# Immunological Relevance of the Coevolution of IDO1 and AHR

**DOI:** 10.3389/fimmu.2014.00521

**Published:** 2014-10-20

**Authors:** Merja Jaronen, Francisco J. Quintana

**Affiliations:** ^1^Center for Neurologic Diseases, Brigham and Women’s Hospital, Harvard Medical School, Boston, MA, USA

**Keywords:** aryl hydrocarbon receptor, 2,3-dioxygenase, tryptophan-2,3-dioxygenase

## Abstract

The aryl hydrocarbon receptor (AHR) is a ligand-activated transcription factor initially identified because of its role in controlling the cellular response to environmental molecules. More recently, AHR has been shown to play a crucial role in controlling innate and adaptive immune responses through several mechanisms, one of which is the regulation of tryptophan metabolism. Indoleamine-2,3-dioxygenase (IDO) and tryptophan-2,3-dioxygenase (TDO) are considered rate-limiting enzymes in the tryptophan catabolism and play important roles in the regulation of the immunity. Moreover, AHR and IDO/TDO are closely interconnected: AHR regulates IDO and TDO expression, and kynurenine produced by IDO/TDO is an AHR agonist. In this review, we propose to examine the relationship between AHR and IDO/TDO and its relevance for the regulation of the immune response in health and disease.

## AHR Signaling Pathways

Aryl hydrocarbon receptor belongs to the family of basic-helix–loop–helix/Per–Arnt–Sim transcription factors. It is abundantly expressed in numerous tissues, such as liver, lung, and placenta (1, 2). Interestingly, AHR is highly conserved through evolution (3), highlighting its importance across the animal kingdom. Originally, AHR was studied in the context of the biological response to environmental toxins such as 2,3,7,8-tetrachlorodibenzo-*p*-dioxin (TCDD). However, it was later found that AHR has an important role in the regulation of immune responses by small molecules provided by the diet, the commensal flora, and metabolism. In its inactive state, AHR resides in the cytosol as part of a complex that includes other proteins such as the 90 kDa heat shock protein (HSP90), the AHR-interacting protein, p23, and the c-SRC protein kinase (4–7). It is thought that HSP90 and p23 protect the receptor from proteolysis and maintain a conformation suitable for ligand binding (8).

Aryl hydrocarbon receptor is activated by ligands binding the PAS-B domain (9), triggering a conformational change that results in the dissociation of AHR from the chaperone proteins and the exposure of its nuclear localization sequence (10). Ligand activation of AHR elicits genomic and non-genomic AHR-dependent signaling pathways. Genomic AHR signaling involves the interaction of AHR with other transcription factors and co-activators to directly regulate the transcription of target genes (7). After ligand activation, AHR translocates to the nucleus where it dimerizes with the AHR nuclear translocator (ARNT) (11) to form an active DNA-binding complex and control the expression of target genes containing xenobiotic response elements (XREs) in their regulatory regions (9). The AHR–ARNT complex can promote or inhibit the expression of its target genes. Moreover, ChIP-seq and microarray studies with different cell types and ligands (12–14) suggest that the AHR target genes in a specific cell are determined by the ligands, and also the identity and developmental stage of the target cells (15).

Non-genomic AHR signaling is more diverse and encompasses, for example, the release of c-SRC from its complex with AHR, resulting in the phosphorylation of c-SRC cellular targets (7). In addition, AHR can promote the degradation of specific target proteins such as estrogen and androgen receptors by the proteasome. This ability to trigger the degradation of specific proteins results from its E3 ligase activity, by which AHR selects proteins for ubiquitination by E2 ubiquitin-conjugating enzymes. The resulting ubiquitinated proteins are then recognized by the 26S proteasome and degraded (16–18). Indeed, following activation AHR itself is eventually exported out from the nucleus and degraded by the 26S proteasome pathway (19–21).

Structure–activity relationship studies showed that AHR’s ligand binding pocket is promiscuous and able to accommodate numerous hydrophobic planar compounds (22). From an historic point of view, AHR can be seen as an endocrine-disrupting chemicals (EDCs) receptor, as it is known that EDCs affect the endocrine system either directly by AHR-dependent changes in gene expression or indirectly via AHR cross-talk with endocrine signaling pathways (23). However, both endogenous and exogenous AHR ligands have been identified. Classical AHR ligands include synthetic aromatic and polycyclic aromatic hydrocarbons (HAHs and PAHs) as well as natural ligands tetrapyrroles, flavonoids, tryptophan derivatives, and dietary carotinoids (24). Interestingly, some of the natural AHR ligands, such as resveratrol (25) and 7-ketocholestrol (26) can act as antagonists rather than agonists. Within the endogenous AHR ligands, tryptophan-derived metabolites have become one of the most interesting and utmost studied group (7). It should be noted that AHR activation in the absence of ligand binding has also been described. Although the relevance of this observation for vertebrates is not completely understood, the ligand-independent activation of AHR might play a physiological role in invertebrates (see below).

## AHR Evolution

Aryl hydrocarbon receptor homologs have been identified in most major groups of animals, including the two main clades of protostome invertebrates as well as deuterostomes (27, 28) highlighting the biological importance of AHR throughout the animal kingdom. AHR homologs identified in invertebrates share similarities with their vertebrate counterparts, such as the interaction with ARNT to recognize XRE (29–31). However, invertebrate AHR homologs do not bind known AHR ligands like TCDD or β-naphthoflavone (29, 31). Indeed, it was recently reported that the metabolic response to xenobiotics in *Caenorhabditis elegans* is not controlled by AHR (32).

In *C. elegans*, the orthologs of AHR and ARNT are encoded by the AHR-related (*ahr-1*) and *ahr-1* associated (*aha-1*) genes, respectively. AHR-1 and AHA have HSP90 binding properties comparable to those of their mammalian counterparts (31). AHR-1 shares 38% amino acid identity with the human AHR over the first 395 amino acids. Furthermore, AHR-1 contains a PAS domain with both PAS-A and PAS-B repeats as well as a bHLH domain where specific residues mediating the recognition of mammalian XREs are conserved (31). However, AHR-1 does not have a glutamine-rich transcriptional activation domain similar to the one present in mammals.

Notably, mutations in AHR-1 affect several aspects of neuronal development determining, for example, the fate of GABAergic neurons in the L1 larval stage, regulating both cell and axon migrations as well as specifying the fate of AVM light touch sensory neuron (33–35). In addition, AHR-1 is involved in social feeding (36), in which nematodes form groups on the border of the bacterial lawn (37).

Recent studies have also demonstrated a role for AHR-1 in regulating the synthesis of long-chain unsaturated fatty acids that eventually produce lipid signaling molecules (38). This finding is consistent with findings in mouse models, where ligand activation of AHR has been linked to alterations in gene expression of fatty acid metabolism (39, 40).

The homologs of mammalian AHR and ARNT are encoded by *spineless* and *tango* in *Drosophila melanogaster* (41, 42). In agreement with observations made in *C. elegans, spineless* does not bind TCDD or β-naphthoflavone (29). In addition, sequence alignments suggest that key residues required for the interaction of mammalian AHR with TCDD are not conserved in *spineless* (29, 41). Thus, although it is still possible that the localization and/or the activity of *spineless* are modified by unknown endogenous ligands, it appears that this protein does not bind classical AHR ligands functional in mammalian systems. Moreover, in certain cells spineless appears to be constitutively active (43). *Spineless* plays a role in several aspects of antenna and leg development (41, 44), photoreceptor cell differentiation (45), and in controlling the morphology of sensory neurons (46).

Not surprisingly, most of our knowledge on mammalian AHR comes from studies on human beings and mice. Key features characterizing mammalian AHR are (1) in contrast to other vertebrates (47) all studied mammalians have a single AHR gene and (2) AHR in mammals is not only involved in the toxic effects of environmental pollutants (48, 49), it also has important roles in development (50–53) and immune responses [reviewed in Ref. (7)]. Indeed, it has been hypothesized that the original function of the AHR might have been developmental regulation and that AHR’s ability to bind HAHs, PAHs, and mediate adaptive responses involving induction of xenobiotic-metabolizing enzymes is a vertebrate innovation (3, 47).

## Kynurenine Pathways TDO/IDO and Immune Regulation

**Figure 1:**
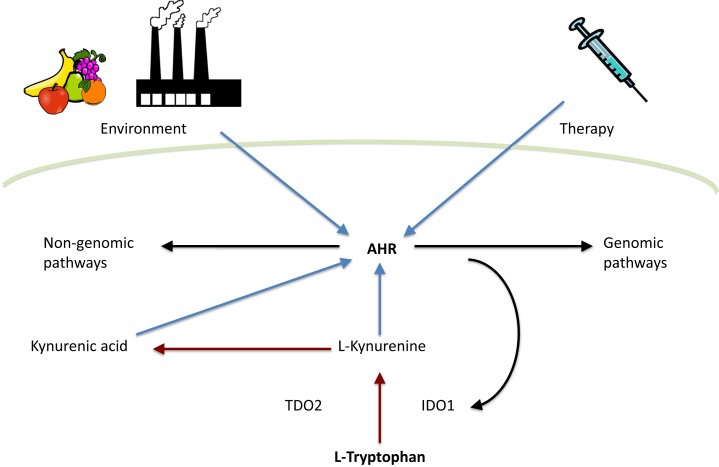
**Interplay between tryptophan metabolism and AHR**. Tryptophan is metabolized by TDO2 and IDO1 to l-kynurenine, which is further converted to kynurenic acid. Both l-kynurenine and kynurenic acid can activate AHR. In addition, AHR activity is influenced by environment and therapy. Finally, AHR can activate either genomic or non-genomic AHR-dependent signaling pathways. Red arrows indicate tryptophan catabolism pathways, blue arrows indicate AHR activation, and black arrows indicate pathways activated by AHR.

Tryptophan metabolites have become one of the most interesting groups of endogenous AHR ligands. Especially kynurenine, an immediate tryptophan metabolite, has been extensively studied in recent years. The metabolic fate of tryptophan is conversion into a range of neuroactive substances, such as serotonin and melatonin. In addition, tryptophan can be catabolized into kynurenine metabolites. Indoleamine-2,3-dioxygenase (IDO1), tryptophan-2,3-dioxygenase (TDO), and recently discovered IDO-related enzyme IDO2 (54) are the first and rate-limiting enzymes converting tryptophan to *N*-formylkynurenine (55, 56) which is then metabolized to l-kynurenine. Both TDO and IDO1 are thought to be intracellular enzymes (57, 58). Therefore, ATP-binding cassette (ABC) transporter (59), enzyme facilitating cellular entry of tryptophan, is considered to be another rate-limiting factor in tryptophan catabolism (60). l-Kynurenine can be catabolized by three different ways: (1) kynurenine monooxygenase, kynureninase, and 3-hydroxyanthranilic acid oxidase catalyze the synthesis of anthranilic acid, 3-hydroxyanthranilic acid, quinolinic acid, and 3-hydroxykynurenine. (2) Kynurenine aminotransfereases catalyze the synthesis of kynurenic acid. (3) Kynurenine monooxygenase and kynurenine aminotransfereases catalyze the synthesis of xanthurenic acid (Figure 1) [reviewed in Ref. (61)].

In human beings, IDO1 is expressed in various tissues and cell subsets following cytokine stimulation during infection, transplantation, pregnancy, autoimmunity, and neoplasia (62–64). IDO1 is constitutively expressed in many human tumors, creating an immunosuppressive microenvironment as a result of tryptophan depletion and the synthesis of immunosuppressive metabolites such as kynurenine (65, 66). Surprisingly, the expression of IDO1 is controlled by AHR (67) via an autocrine AHR-IL6-STAT3 signaling loop (68). In addition, tryptophan starvation caused by IDO1 activity, together with IDO1-dependent tryptophan catabolism, inhibits the proliferation and activation of antigen-specific T lymphocytes and induces immune tolerance (69–72). In addition, strong evidence suggests that tryptophan catabolism can inhibit T-cell based adaptive immunity by inducing the differentiation of regulatory T cells (Treg) in tumors (62, 73–75). Interestingly, kynurenine is also indicated to promote the differentiation of Tregs (76) while suppressing antigen-specific T-cell responses (77).

In mammals, TDO2 is expressed primarily in the liver (78–80) but can also be detected in other tissues such as the brain (79, 81–83). TDO2 is constitutively expressed and activated in gliomas (84). Recently, lipopolysaccharide was demonstrated to induce TDO2 expression and via consequent production of kynurenine activate AHR-dependent pathways leading to protection against endotoxin challenge (85). In addition, this study also reported that endotoxin tolerance is also mediated by AHR as it was demonstrated that AHR activation by kynurenine elicits the c-SRC dependent phosphorylation of IDO1, which further regulates TGFβ1 production by dendritic cells as well as limits immunopathology triggered by both *Salmonella typhimurium* and group B *Streptococcus* (85). Furthermore, TDO2 derived kynurenine has been demonstrated to suppress antitumor immune responses as well as promote survival and motility of tumor cells via AHR in an autocrine manner (84). Note that kynurenic acid can also activate AHR signaling (86).

## IDO/TDO Evolution

Unfortunately, not much is know about the kynurenine pathway in nematodes. However, the study of intestinal autofluorescence in relation to tryptophan catabolism revealed that nematodes having a mutated *flu-1* gene show altered gut granule autofluoresence as well as decreased kynurenine hydroxylase activity (87). Whereas, *flu-2* mutants have reduced kynureninase and gut granule autofluorescence (87). In support of these observations, the *C. elegans* genome has homologs of kynurenine hydroxylase and kynureninase in the vicinity of *flu-1* and *flu-2* loci (88).

**Figure 2:**
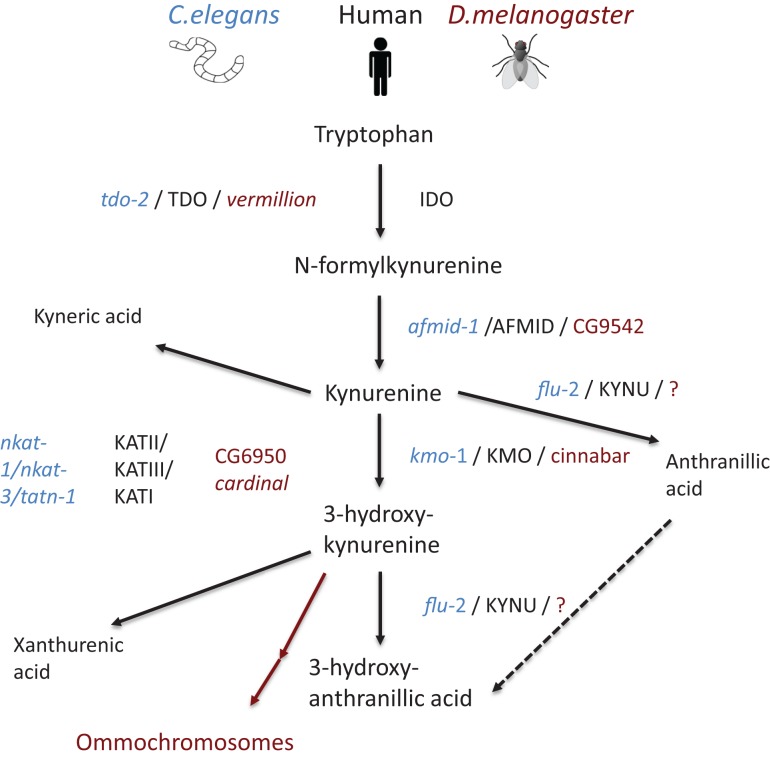
**Schematic representation of the kynurenine pathway in human beings (mammal), *C. elegans*, and *D. melanogaster***. Pathway tree demonstrates differences between mammalian and invertebrate tryptophan catabolism. Mammalian enzymes are depicted in black, *C. elegans* enzymes in blue, and *D. melanogaster* in red. Modified from Ref. (89)

Additional putative kynurenine pathway related genes have been identified in the *C. elegans* genome (89) (Figure 2). The knock down of *tdo-2*, for example, abrogated the gut granule fluorescence (90, 91). Involvement of the *C. elegans* kynurenine pathway has been demonstrated in neurodegeneration and aging: in a *C. elegans* model of Parkinson’s disease; RNAi knock down of *tdo-2* reduced α-synuclein aggregation-induced toxicity and increased life span (92). However, these effects were proven to be a result of increased tryptophan rather than changed levels of kynurenines (92).

In *D. melanogaster*, tryptophan catabolism takes place in pigmented eyes (93–95). Remarkably, the role of kynurenine pathway in eye function is conserved from flies to mammals, as it plays an essential role in protecting the lens from ultraviolet irradiation (96). *D. melanogaster* TDO2 is encoded by *vermillion*. Flies having the *vermillion* mutation lack brown pigment in their eyes and have been thought to be deficient for TDO2 activity (93, 94, 97, 98). This was verified when kynurenine pathway and related genes were described in full in 2003 (99). In the same way, as in *C. elegans*, loss of *vermillion* function has been demonstrated to be neuroprotective in *D. melanogaster* model of Huntington’s disease (100). In addition, loss of *vermillion* function extend the life span of *D. melanogaster* (101, 102) while resulting gradual memory decline (103). Furthermore, white eye mutants having impaired ABC transport show extended life spans (102). In addition, other *D. melanogaster* mutants, cardinal and cinnabar, resulting in excess of 3-hydroxykynurenine and neuroprotective kynurenic acid, have been demonstrated to modify the brain plasticity (104).

## Conclusion

Aryl hydrocarbon receptor, a member of the dHLH-PAS superfamily, has been identified both in invertebrates and vertebrates, suggesting that the ancestral AHR gene arose over 500 million years ago (3). In vertebrates, especially in mammals, the activity of AHR is mostly regulated by its interactions with ligands. However, in invertebrates (e.g. *C. elegans*) AHR does not seem to interact with TCDD or any other known ligand (105), and it is constitutively localized in the nuclei of certain cells suggesting ligand-independent activation (34). Similar observations have been made for *D. melanogaster’s spineless* (29). Although one cannot rule out the possibility that invertebrates require a different kind of AHR ligands than vertebrates, it has been speculated that in early metazoans AHR might have had a ligand-independent roles in development. Thus, the ability of AHR to interact with ligands, bind HAHs and PAHs, and regulate xenobiotic-metabolizing enzymes has been postulated to be a vertebrate novelty (3, 47).

Aryl hydrocarbon receptor signaling modulates development and immune function in mammals (7). Fairly recently, the involvement of tryptophan metabolism has been implicated in regulating both innate and adaptive immune responses. Most importantly, kynurenine produced by TDO or IDO1 during tryptophan catabolism has been identified as an AHR ligand, linking IDO/TDO to AHR. Considering the evolutionary conservation of the kynurenine pathway, it is tempting to speculate that the cross-talk between AHR and IDO/TDO immunoregulatory pathways is a recent evolutionary innovation aimed at providing a mechanism to fine tune the immune response in response to environmental cues provided by the tissue microenvironment. This interpretation suggests that approaches targeting both AHR and IDO/TDO are likely to provide efficient new avenues for the therapeutic manipulation of the immune response.

## Conflict of Interest Statement

The authors declare that the research was conducted in the absence of any commercial or financial relationships that could be construed as a potential conflict of interest.
